# Elements of Transcriptional Machinery Are Compatible among Plants and Mammals

**DOI:** 10.1371/journal.pone.0053737

**Published:** 2013-01-11

**Authors:** Annette Wolf, Nina Akrap, Berenice Marg, Helena Galliardt, Martyna Heiligentag, Fabian Humpert, Markus Sauer, Barbara Kaltschmidt, Christian Kaltschmidt, Thorsten Seidel

**Affiliations:** 1 Dynamic Cell Imaging, University of Bielefeld, Bielefeld, Germany; 2 Biomolecular Photonics, University of Bielefeld, Bielefeld, Germany; 3 Biotechnology & Biophysics, Biozentrum, Würzburg University, Würzburg, Germany; 4 Molecular Neurobiology, University of Bielefeld, Bielefeld, Germany; 5 Department of Cell Biology, University of Bielefeld, Bielefeld, Germany; Beijing Institute of Microbiology and Epidemiology, China

## Abstract

In the present work, the objective has been to analyse the compatibility of plant and human transcriptional machinery. The experiments revealed that nuclear import and export are conserved among plants and mammals. Further it has been shown that transactivation of a human promoter occurs by human transcription factor NF-κB in plant cells, demonstrating that the transcriptional machinery is highly conserved in both kingdoms. Functionality was also seen for regulatory elements of NF-κB such as its inhibitor IκB isoform α that negatively regulated the transactivation activity of the p50/RelA heterodimer by interaction with NF-κB in plant cells. Nuclear export of RelA could be demonstrated by FRAP-measurements so that RelA shows nucleo-cytoplasmic shuttling as reported for RelA in mammalian cells. The data reveals the high level of compatibility of human transcriptional elements with the plant transcriptional machinery. Thus, *Arabidopsis thaliana* mesophyll protoplasts might provide a new heterologous expression system for the investigation of the human NF-κB signaling pathways. The system successfully enabled the controlled manipulation of NF-κB activity. We suggest the plant protoplast system as a tool for reconstitution and analyses of mammalian pathways and for direct observation of responses to e.g. pharmaceuticals. The major advantage of the system is the absence of interference with endogenous factors that affect and crosstalk with the pathway.

## Introduction

A first hint for transcriptional compatibility of plants and mammals was given by Gitzinger and co-workers, who showed that human promoters can drive protein expression in moss protoplasts [1]. In this report we investigated the activity of a human transcription factor and its target promoter in *Arabidopsis thaliana* mesophyll protoplast. The experiments aimed at identification of a novel heterologous expression system for the investigation of human signal transduction pathways in particular for analysing the human NF-κB (Nuclear Factor kappa light chain enhancer in B cells) pathway. Members of the NF-κB family serve as key regulators of the immune and inflammatory response, apoptosis, cell proliferation and differentiation [2], [3], [4]. The dimeric form of NF-κB represents the active conformation and NF-κB could exist as a homodimer or heterodimer [5]. All members share a Rel Homology Domain (RHD), which mediates regulation, dimerization and DNA binding. NF-κB subunits containing a transcriptional activation domain (TAD) could activate gene transcription (class II, RelA), whereas homodimers of Class I proteins without a TAD (p50) could repress transcription by blocking the κB binding sites [6]. The most prominent NF-κB complex is the p50/RelA heterodimer. The activity of the NF-κB dimer is mainly regulated by the non covalent interaction with the inhibitory protein (IκB) which masks the Nuclear Localization Signal (NLS) of the NF-κB heterodimer. IκB sequesters the NF-κB dimer in the cytoplasm of unstimulated cells [7]. Upon adequate stimulus (LPS, TNF-α, PMA), kinases, such as NIK (NF-κB-inducing kinase) or members of the MEKK family, activate the IKK (IκB Kinase) complex [8]. The IKKs phosphorylate two conserved serine residues in the N-terminal domain of IκB, which leads to ubiquitination and therefore, to rapid degradation of IκB by the proteasome [9]. In consequence, the heterodimer is released and enters the nucleus. Here, it activates the transcription of target genes [9]. Newly synthesized IκBα accumulates in the cytoplasm directly after NF-κB induction. Subsequently, unbound IκBα enters the nucleus removing NF-κB from target genes and shuttling it back to the cytosol [10]. Thus, the NF-κB dependent signal is terminated. Similar to NF-κB IκB belongs to a family of structurally similar proteins with distinct binding preferences towards the individual NF-κB complexes [11], [12].

We successfully reconstituted parts of the canonical NF-κB pathway (including p50/RelA heterodimer and their inhibitory protein IκB) in plant protoplasts. If compared to human HEK293 cells, the proteins of the NF-κB pathway retained their special localization, interactions and function in plant cells. The plant expression system showed a defined response with respect to actively introduced components of the signal transduction, whereas the concentration and presence of the endogenous components and their impact on the promoter activity is widely unknown in human cell lines.

## Materials and Methods

### Materials

*A. thaliana* (Columbia Col-0) were grown in soil-culture in a growth chamber with 12 h light (240 µmol quanta m^−2 ^s^−1^, 19°C) and 12 h dark (18°C) with 60% relative humidity. For protoplast isolation *A. thaliana* leaves were harvested from soil grown plants at the age of about four weeks. HEK293FT-cells (Invitrogen) were grown in DMEM-Medium with 4.5 g L^−1^ glucose and stable glutamine (PAA) containing 10% fetal bovine serum at 37°C with 5% CO_2_. Transfection using PromoFectin (PromoKine) was performed with 1.5 µg plasmid DNA according to the Manufactor’s instruction. Cells were cultured on 60 mm cell culture dishes coated with gelatine 24 h prior to transfection.

### Protein Expression

The fluorescent proteins His_6_-ECFP and His_6_-ECFP-EYFP were heterologously expressed in *Escherichia coli* strain BL21. The His_6_-tagged proteins were purified by Ni–NTA-affinity chromatography and finally dialysed against 40 mM phosphate buffer (pH 7).

### Methods of Molecular Biology

The coding sequences of p50, RelA and IκB were amplified by PCR and flanking restriction sites were introduced by PCR those allow for insertion into the plasmids 35S-ECFP-NosT, 35S-EYFP-NosT, 35S-Dronpa-NosT (RelA, IκB; BamHI & AgeI) and 35S-ECFP-C, 35S-EYFP-C (p50; NotI & EcoRI). Based on pEGFP-N1 (Clontech) the vectors CMV-ECFP, CMV-EYFP and CMV-mCherry were generated by replacing the coding sequence of EGFP with the coding sequences of ECFP, EYFP, mCherry, respectively. Subsequently p50, RelA and IκB were inserted into these vectors using HindIII and AgeI restriction sites. Vectors for BiFC were generated by replacing the fluorophore in the plasmid 35S-EYFP-NosT by coding sequences that encode for the fragments 1–155 and 156–238 of EYFP, respectively. RelA was inserted using BamHI and AgeI restriction sites. For transactivation assays, the Ikba-promoter was placed upstream the CaMV35S TATA-box in the plasmid 35S-YFP-NosT. Furthermore, plasmids were generated that enable the expression of untagged RelA and IkB in plant cells, using primers adding flanking BamHI and AgeI restriction sites and maintaining the stop codon. For p50, mCherry has been removed from 35S-mCherry-p50 by double digestion with XmaI and BsrG1, followed by fill-in reaction with Klenow-fragment and blunt end ligation.

### Isolation and Transfection of Mesophyll Protoplasts

The isolation and the polyethylene glycol mediated transfection of *A. thaliana* protoplasts were performed as described before [13].

### Fluorescence Microscopy

Fluorescence microscopy was performed with an upright microscope Zeiss Axioskop2 with 63-fold magnification (Zeiss Achroplan 63x/0.95W). For the detection of CFP, YFP and Hoechst-staining the filter sets 47, 46 and 01 were applied, respectively. Images were obtained with a Zeiss AxioCam ICc1 camera controlled by the Zeiss Axiovision Rel. 4.7 software.

### Confocal Laser Scanning Microscopy

For image acquisition, a Leica TCS SP2 confocal system with 40-fold magnification (water immersion objective HCX APO L 40x/0.8W UVI, NA = 0.8) was used. The scan speed was 400 Hz, the image resolution 1024*1024 pixels and 12 bit scanning mode was chosen to improve the signal to noise ratio. ECFP was detected in the range of 470–510 nm, EYFP-emission was detected in the range of 530–600 nm and chlorophyll autofluorescence in the range of 650–700 nm. The settings for ECFP- and EYFP-detection were also applied for BiFC.

For FRET-analyses the FRET signal and the EYFP-reference channel were recorded with photomultiplier 3 between 530 and 600 nm. The ECFP emission was detected with photomultiplier 2 between 470 and 510 nm. A 458 nm excitation was applied to record ECFP and FRET-emission, 514 nm for excitation of EYFP. For FRET-measurements the double dichroic mirror DD458/514 was used. The scan speed was 400 Hz, the image resolution 1024 · 1024 pixels, the pinhole diameter 100 µm. For each set of transformation, images from more than 20 cells were obtained, and each experiment was repeated independently [14].

The correction factors α (0.88) and β (0.64) were determined with cells expressing solely EYFP and ECFP, respectively. The correction factor ξ was estimated to be 0.53:
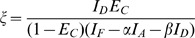



The required true FRET-efficiency E_c_ of an ECFP-EYFP fusion protein was determined *in vitro* before by recording the alteration of donor emission relatively to emission of similar molar amount of solely ECFP and was found to be 0.46. According to Beemiller et al. [15] the FRET-efficiency E was defined as:
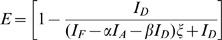



To investigate transactivation of IκB-promoter by NF-κB *in planta* ECFP-reference was detected in the range of 470–510 nm using excitation at 458 nm and the transactivation-derived EYFP-signal in the range of 530–600 nm with 514 nm excitation. The ratio of EYFP-emission I_YFP_ and ECFP-emission I_CFP_ was defined as transactivation activity R_ta_:
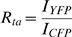



The detection of protein-protein interactions by BiFC was performed according to the transactivation, since EYFP folding was recorded and normalized to ECFP emission.

### FRAP-analysis

FRAP-analysis was performed according to Lummer et al. [16].

## Results and Discussion

### Subcellular Localisation of NF-κB Subunits p50 and RelA is Identical in Protoplasts and HEK293 Cells

**Figure 1:**
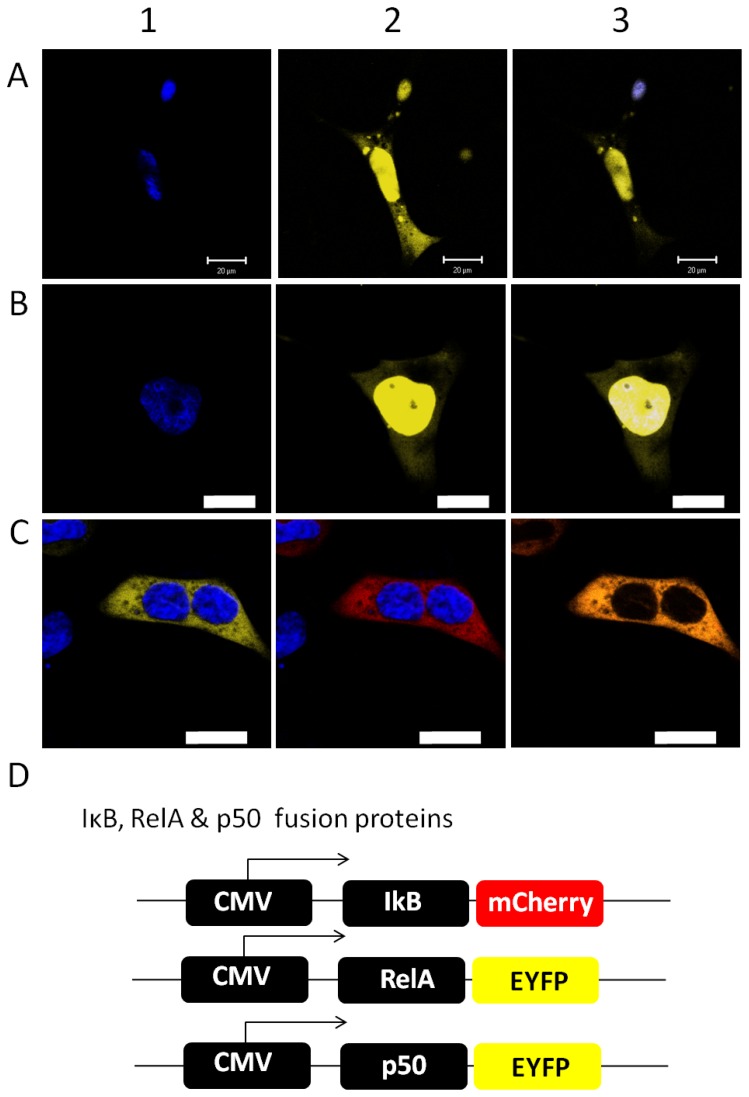
Localization of p50, RelA and IκB in HEK293 cells. The fusion proteins (see D) for constructs) were expressed in HEK293 cells, nuclei were stained with Hoechst33342 and images were obtained by confocal laser scanning microscopy. Representative images are shown. Scale bars represent 20 µm. A, B) Co-localisation of NF-κB subunits p50 (A2) and RelA (B2) and nuclear Hoechst-staining, respectively (A1, B1). Overlays of both channels are shown in A3 and B3). C) Effect of IκB on the localisation of RelA in HEK293 cells. Hoechst staining has been applied to visualize nuclear localization as reference for the RelA/IκB co-localization. The Hoechst33342-stained nuclei are shown in blue. RelA-EYFP (B1) and IκB-mCherry (B2) were co-expressed and orange colour in the merged image demonstrates co-localization (B3). C) Overview of the applied constructs under control of the CMV-promoter.

**Figure 2:**
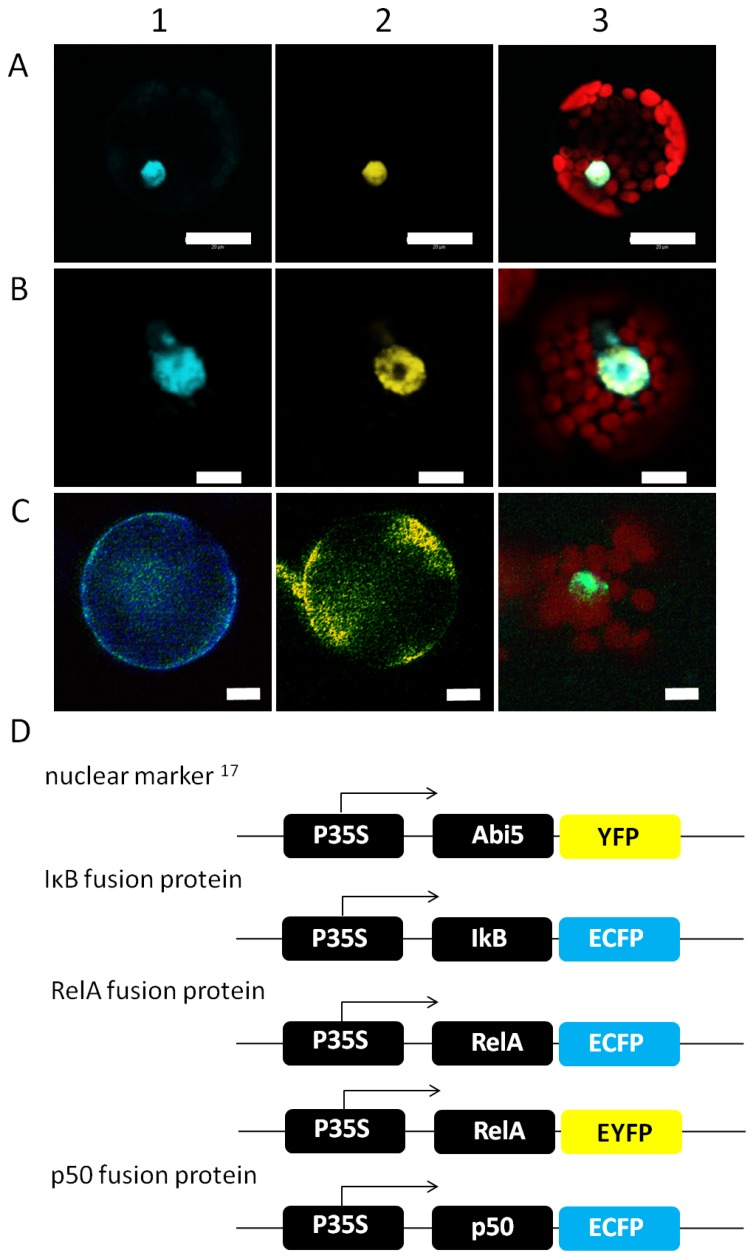
Subcellular Localization of p50, RelA and IκB in plant cells. A, B) Co-localisation of the NF-κB subunits p50 (A) and RelA (B) with the plant transcription factor Abi5 (Lopez-Molina et al. 2003). The fusion proteins were expressed in Arabidopsis protoplasts and detected by confocal laser scanning microscopy. Abi5 was fused to YFP (A2, B2 yellow), the NF-κB subunits to ECFP (A1, B1 cyan). Plastid-derived autofluorescence is shown in red in the overlay images (A3, B3). C) Effect of IκB on the localisation of RelA in plant cells observed by fluorescence microscopy. The localisation of IκB is shown in cyan (C1), yellow staining has been chosen for the localization of RelA (C2) and the image (C3 shows Hoechst staining (green) of the nucleus as well as the chlorophyll autofluorescence in red. In (C) RelA and IκB are not co-localized with nuclear staining. Representative images are shown. Scale bars correspond to 20 (A) and 10 µm (B, C). D) Construct design of Abi5-YFP, IκB-ECFP, p50-ECFP and RelA-EYFP under control of the CaMV35S-promoter.

**Figure 3:**
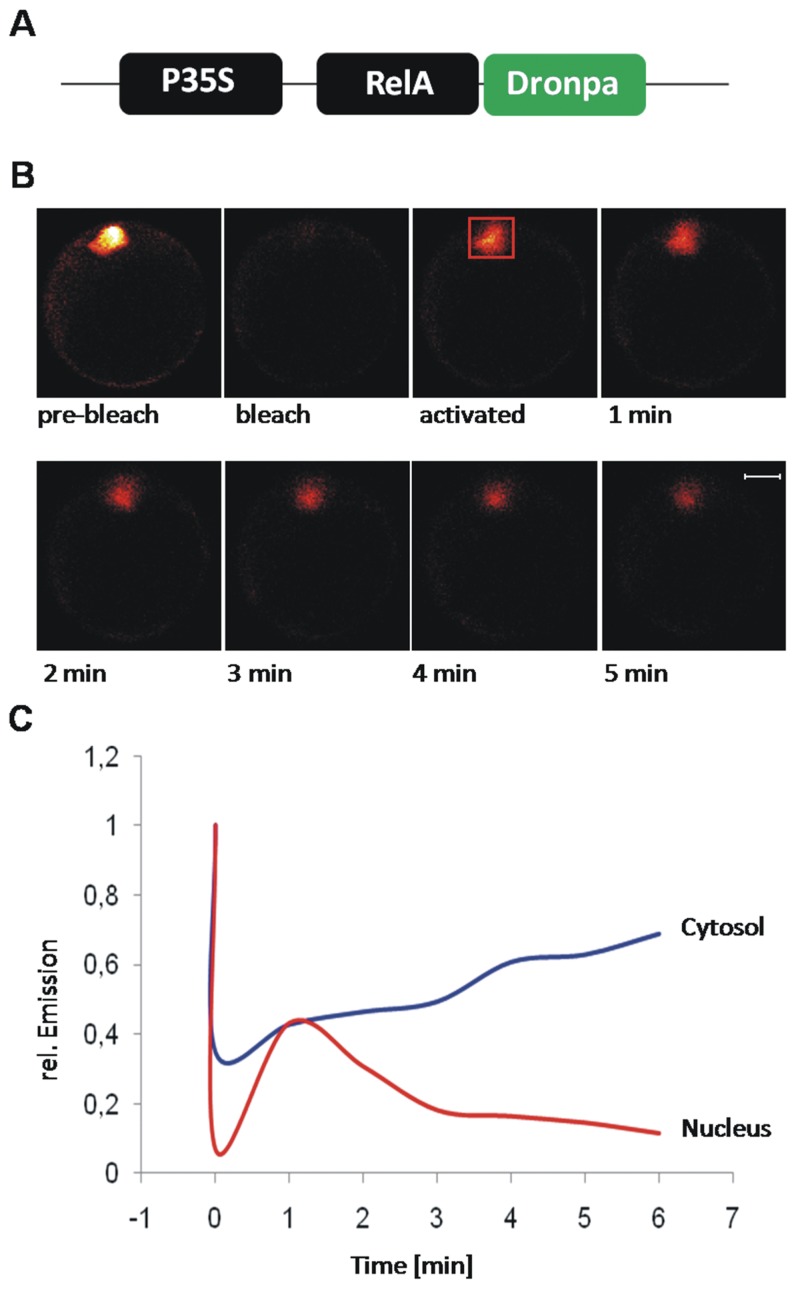
FRAP-analysis of RelA nuclear export. A) Fusion construct for the analysis of nuclear export in plant cells. RelA is fused to Dronpa via its C-terminus. Figure B) shows representative images for pre-bleach, bleaching, activation and diffusion. C) The fluorescence emission intensity of Dronpa in the cytosol (blue) and the nucleus (red) is plotted against the time. Bleaching was performed at time point “0”, diffusion was monitored for five minutes post activation.

Initially, the subcellular localization of NF-κB and IκB subunits has been comparatively analysed in human and plant cells. The NF-κB subunits p50 and RelA were transiently expressed as ECFP-fusion proteins in *A. thaliana* mesophyll protoplasts as well as in HEK293 cells and the localization has been analyzed by confocal laser scanning microscopy with a Leica TCS SP2 system. As expected, p50 and RelA were imported into the nucleus in human cells, the nucleus has been stained with Hoechst33342 for identification. NF-κB subunits were detectable in the cytosol, if the signal amplification was increased resulting in oversaturation of the nuclear derived emission (Fig. 1). In plant cells, the subcellular localisations of p50 and RelA were analysed in cells co-expressing the plant transcription factor Abi5 fused to YFP, respectively [17]. Abi5 localises to the nucleus in the plant cell and served as compartment marker [17]. Both NF-κB subunits were found co-localised with the Abi5-YFP fusion protein in the nucleus of the plant cell (Fig. 2). Similar to mammalian cells, RelA can be observed in the cytosol if the nuclear emission is oversaturated due to increased sensitivity of the detector (Fig. 3B, pre bleach). This demonstrates the ability of the plant cell to recognize the human nuclear localisation signal and to finally import NF-κB. In contrast, IκB-ECFP showed an equal distribution in cytosol and nucleus in both cell types (Fig. S1) resembling previous observations in mammalian cells [18]. In accordance with the analysis of RelA, the nuclear fraction of IκB was identified by co-localisation with Hoechst33342-staining in HEK293-cells and with Abi5 in plant protoplasts (Fig. 2, Fig. S1). The IκB isoform α negatively regulates the p50/RelA heterodimer by capturing NF-κB in the cytosol [7]. Co-expressing RelA-EYFP and IκB-ECFP, RelA and IκB were visible in the cytosol in both cell types, demonstrating that overexpression of IκB efficiently traps RelA in the cytosol and prevents its import into the nucleus in plant and human cells (Figs. 1 and 2).

### RelA Shows Nucleo-cytoplasmic Shuttling in Arabidopsis Protoplasts

A central requirement for full NF-κB functionality is its ability to enter the nucleus and to be exported again. It should be noted that RelA bears a nuclear export signal that is inactivated in the presence of p50 [19]. Therefore, the dynamic of the nucleo-cytoplasmatic shuttling of RelA was analysed by FRAP/FLIP-analysis with the photoactivalable fluorescent protein Dronpa and a single photon sensitive confocal microscope [16]. The experiments were performed in Arabidopsis protoplasts that were transiently transfected with RelA-Dronpa. Initially, Dronpa was completely bleached by intensive 488 nm (argon ion laser) illumination of the observed cell. In the next, Dronpa was activated by illumination of the nuclear area with 404 nm (diode laser) and the emission was recorded all over the cell to monitor nuclear export by combined FRAP/FLIP-analysis. After activation, an increase of the cytosolic emission intensity was observed within the next two minutes that co-occured with a reduction of fluorescence emission in the nucleus (Fig. 3). The half time of fluorescence loss in the nucleus was approximately two minutes. The increase of cytosolic emission intensity at the expense of the nuclear fluorescence demonstrates the increasing amount of p65-Dronpa in the cytosol accompanied by its disappearance in the nucleus and thus, the nuclear export of p65-Dronpa in the plant cell. In mice, NF-kB signal termination by nuclear export of RelA occurred within a couple of minutes [20] and is in good agreement with the time scale of nuclear export of RelA in the plant cell. Nuclear pore complexes (NPC) are required for nuclear import and export and NES-mediated transport is typically facilitated [21]. The diameter of NPC has been reported to be 2.6 nm so that proteins exceeding 40 kDa require facilitated transport [22], [23]. Furthermore, a high number of proteins that form nuclear pore complexes and take in part in transport across the NPC are conserved among plants and mammals [24]. Hence, it can be concluded, that RelA shows nuclear cytoplasmic shuttling, by an active transport mechanism similar to its reported behaviour in human cells [25]. The nucleocytoplasmic shuttling of RelA in the plant cell might not be an exceptional case of compatibility among the plant and mammalian nuclear import/export machinery. This thesis is supported by the work of Ziemienowicz et al. (2003) who demonstrated functionality for the plant transportin 1 in HeLa cells and their analyses further revealed its interaction with human hnRNP A1 and yeast Nab2p, respectively, as well as nuclear import of the plant RNA-binding protein AtGRP7 in HeLa cells [26].

### RelA and p50 Dimerize and Form NF-κB that Interacts with IκB

The active form of NF-κB is a dimeric arrangement. The functionality of NF-κB depends strongly on the type of dimerisation and participating subunits e.g. RelA and p50. Furthermore, the inhibitory interaction with IκB is crucial for the regulation of NF-κB’s activity. Therefore, the interaction between NF-κB subunits has been analysed by Förster resonance energy transfer (FRET)-analyses and bimolecular fluorescence complementation (BiFC)-assays in plant cells applying confocal laser scanning microscopy.

**Figure 4:**
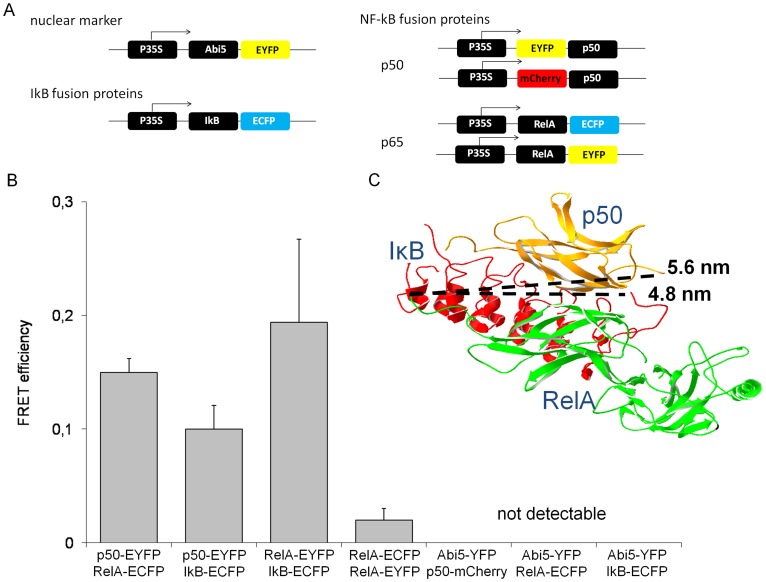
FRET-analysis of NF-κB subunits and IκB. A) Fusion constructs that have been applied for FRET-measurements. B) The FRET-efficiency between the FRET-pairs p50-ECFP/RelA-EYFP, p50-ECFP/IκB-EYFP, RelA-ECFP/IκB-EYFP, p50-ECFP/Abi5-YFP, RelA-ECFP/Abi5-YFP and IκB-ECFP/Abi5-YFP was measured in more than 40 plant cells for each combination. The data represent mean average ± S.E. C) Crystal structure of NF-κB/IκB trimer and the distances between RelA-C-terminus and p50-N-terminus or IκB-C-terminus, respectively. The crystal structure was obtained from RCSB protein data bank, accession number 1IKN [31].

**Figure 5:**
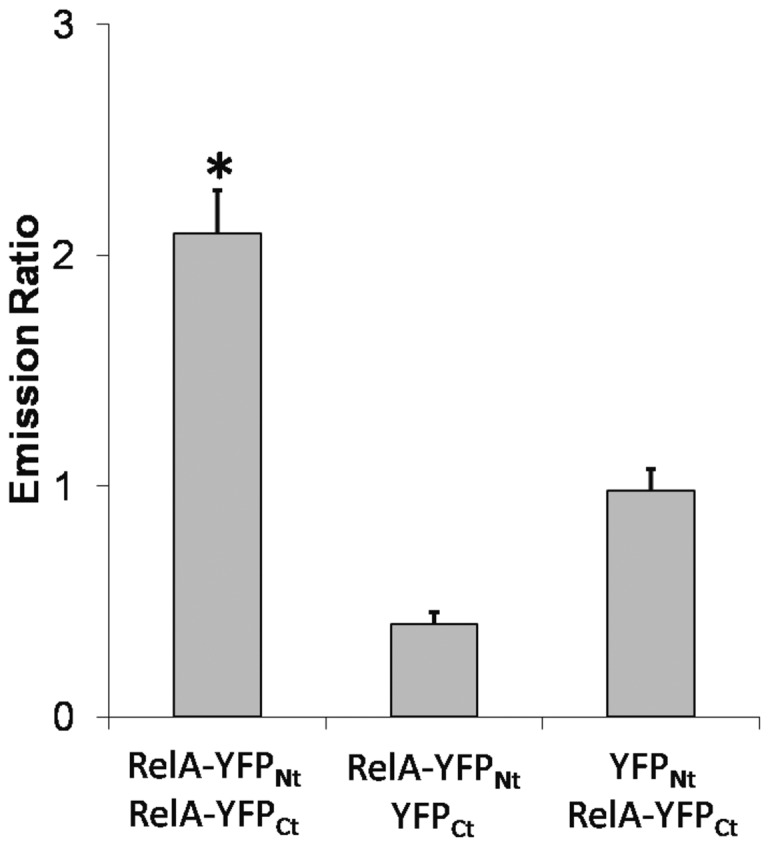
Homodimerization of RelA. The homodimerization of RelA has been analysed by bimolecular fluorescence complementation in living plant protoplasts. The EYFP emission was recorded in 40 cells and normalized to ECFP-emission. Mean average ± S.E. is shown. The combinations RelA-YFP_Nt_/YFP_Ct_ and YFP_Nt_/RelA-YFP_Ct_ served as control for occasional folding of YFP. Asterisk marks significant difference in EYFP-folding between the sample for RelA-homodimerization and the controls. Student’s t-test has been applied (p = 7,2E-11 for RelA-YFP_Nt_/YFP_Ct_ and p = 2,6E-6 for YFP_Nt_/RelA-YFP_Ct_).

FRET-efficiency has been measured for the protein combinations p50/RelA, RelA/IκB and the RelA-homodimer. Significant FRET of 0.15±0.01(Mean ± S.E., n = 22) was observed between p50 and RelA, for homodimerization of RelA the FRET-efficiency was 0.02±0.01 (Mean ± S.E., n = 69) and hence, does not provide evidence for efficient homodimerization of RelA (Fig. 4). However, the FRET-efficiency of homodimerization is close to the FRET-efficiency that has been published for RelA-dimers in HeLa cells [27]. For RelA and IκB the FRET-efficiency was 0.19±0.04 (Mean ± S.E., n = 67; Fig. 4). The FRET-efficiencies between NF-κB-subunits or IκB and the plant transcription factor Abi5 were obtained as control for unspecific interactions in the nucleus, respectively. In these cases an energy transfer was not detectable. For quantification of EYFP-based BiFC-experiments the EYFP fluorescence emission was recorded in the plant cell and related to the emission of ECFP that was expressed under control of the CaMV35S-promoter as standard for the protein expression capacity of the analysed cells. Combination of the constructs RelA-EYFP_Nt_ and EYFP_Ct_ as well as RelA-EYFP_Ct_ and EYFP_Nt_ served as negative controls for occasional background fluorophore formation. The BiFC-experiments with the constructs RelA-EYFP_Nt_ and RelA-EYFP_Ct_ revealed the formation of RelA-homodimers in Arabidopsis protoplasts and showed significant increase of relative EYFP emission in comparison to the negative controls (Fig. 5).

The results showed the dimerisation of p50 and RelA and confirmed the interaction between IκB and RelA in the plant cells, an interaction that has been indicated by the altered subcellular localisation of RelA in the presence of IκB before. Furthermore, the homo-dimerisation of RelA was not proven by FRET but by BiFC. The data confirmed the low affinity of RelA homodimerization and the preference for the p50/RelA heterodimer, as it has been shown before by *in vitro* measurements [28].

### Transactivation of IκBα-promoter by NF-κB

**Figure 6:**
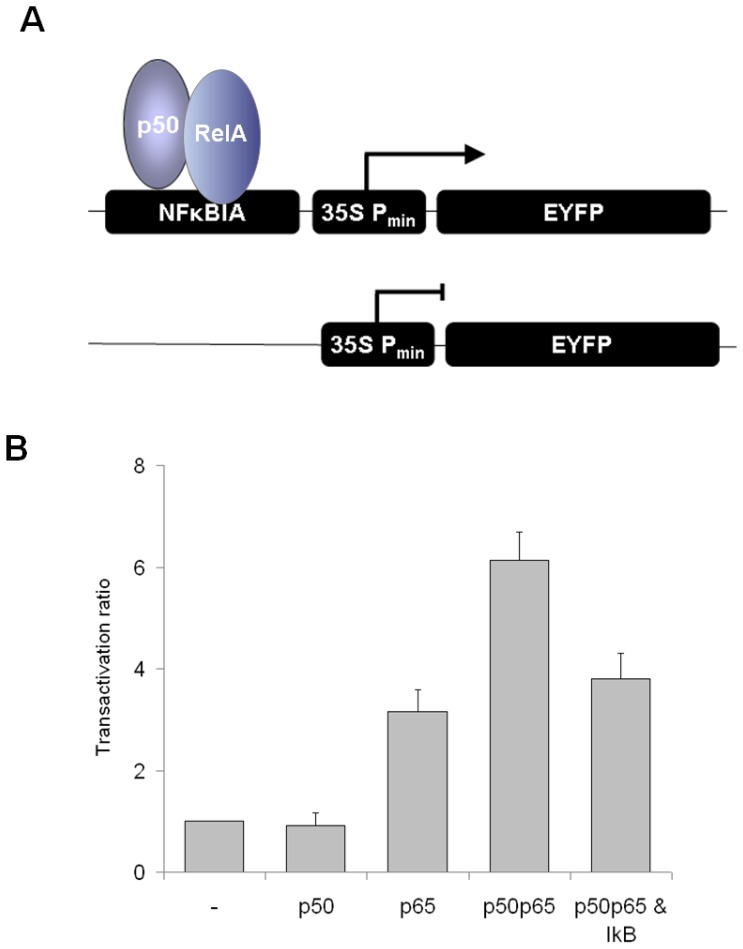
Transactivation of the IκBα promoter. The expression of EYFP is driven by the IκBα promoter (NFκBIA), ECFP is constitutively expressed as reference. The IκBα promoter was induced by CaMV35S-driven expression of p50/RelA, p50, RelA and p50/RelA/IκB, respectively. A) Scheme of transactivation by p50/RelA homodimer B) Transactivation ratio is given as ratio of EYFP and ECFP emission, normalized to the activity of the IκBα promoter that has been set to 1.0. The transactivation potential has been analyzed for p50, RelA, p50/RelA and p50/RelA in the presence of IκB. The mean ± S.E. of more than 20 cells is shown. Student’s t-test has been applied to compare the transactivation with the activity of the IκBα promoter (p = 0.346 for p50, p = 0.0241E-11 for p65, p = 4.012E-08 for p50/RelA and p = 3.739E-07 for p50/RelA/IκB). Inhibition of NF-κB by IκB was highly significant (p = 3.041E-05).

The remaining question has been, if a human promoter can be transactivated by a human transcription factor in the plant cell, demonstrating that it is recognized by the transcriptional machinery and thus, is active in the plant cell. Therefore, the IκBα-promoter was placed upstream the CaMV35S TATA-box and drove the expression of EYFP, representing the reporter construct. Expression of ECFP under control of the CaMV35S full length promoter (control plasmid CaMV35S-ECFP-NosT) served as marker for transfected cells and for compensation of variable protein expression capacity. The promoter activity is given as ratio of EYFP and ECFP emission intensities and cells were analysed by confocal laser scanning microscopy using a Leica TCS SP2 system. The basal EYFP-expression was similar for the CaMV35S minimal promoter and the combination of CaMV35S minimal promoter and IκBα-promoter. The IκBα-promoter was selected for the analysis of the NFκB induced transactivation potential in plant cells due to its three κB-sites with a high affinity for p50/RelA heterodimers and because it is well characterized and the activity is rapidly changed after activation of NF-κB [29]. Furthermore, the activation of the IκBα-promoter represents an important part of the IκB-NFκB feedback loop, finally resulting in IκB-mediated autoregulatory termination of NFκB-signaling. To this end, IκBα represents a target of NF-κB [5]. The NF-κB sites differ in their affinity to NF-κB subunits. Both homodimers of p50 and RelA as well as the heterodimer bind to NF-κB sites 2 and 3 in the promoter. NF-κB site 1 can be occupied by the p50 homodimer and the NF-κB heterodimer. The binding sites are known to cooperatively interact during activation of the IκBa-promoter [29]. Basically, the cells were co-transfected with the reporter construct and the control plasmid. CaMV35S-driven expression of RelA resulted in approximately fourfold increase of EYFP emission. The increase of EYFP-emission was more than sixfold due to transactivation by the heterodimer p50/RelA (Fig. 6). Obviously the RelA homodimer which binds to binding sites 1 and 2 is less efficient in transactivation of the IκB-promoter than the heterodimer which binds to all three binding sites, demonstrating the cooperativity of the binding sites. In the presence of IκB, the transactivation by the heterodimer dropped to 3.5-fold relative emission in comparison to the basal activity, but transactivation was not totally inhibited (Fig. 6). This might be caused by a reduced expression level or less stability of IκB in comparison to the NF-κB heterodimer. It cannot be discriminated whether IκB inhibits transactivation by retaining NF-κB in the cytosol or by disturbing NF-κB/DNA interactions through its PEST-sequence [30]. Nevertheless, the data provides an additional proof that nuclear import of p50 and RelA occurs and recognition of human NLS is enabled by the plant nuclear import machinery. Whereas the N-terminal Rel homology (Rh)-domain of the NF-κB subunits is responsible for DNA-binding and dimerisation, the C-terminal transactivation (TA)-domain of Rel-proteins is responsible for transactivation [7]. Obviously, both domains are functional in the plant cell as indicated by RelA-induced EYFP-expression and further enhanced expression in presence of the heterodimer consisting of p50 and RelA.

### Conclusions

The data demonstrates that human transcription factors are functional in plant protoplasts on the levels of subcellular localisation, complex formation and transcriptional activation and regulation. It can be concluded, that the nuclear import and export as well as induction of transcription by transcription factors are conserved among plants and mammals, although full functionality might not be given for any human transcription factor in the plant cell. The NF-κB family is not conserved between mammals and plants, so that the plant cell lacks Rel-proteins as well as IκB isoforms. So the application of protoplasts is not limited to the simple expression of human proteins. The protoplasts expression system allows for reconstitution of elements of the NF-κB-pathway without interference with the endogenous components of NF-κB signalling. The presence of endogenous NF-κB and IκB and the crosstalk of multiple pathways that are integrated at the stage of NF-κB represent an error source that is hard to control in human cells, since the actual protein amount and its activity remains elusive. The same is true for any other factor that affects NF-κB activity in human cells as the cell-type specific and developmental regulated influence of e.g. protein kinase A or the peroxiredoxin 1 on NF-κB activity cannot be ruled out in transactivation assays so that the crosstalk of several pathways has to result in datasets that cannot be discussed and ascribed to a defined pathway anymore. Therefore, the plant protoplasts represent a promising expression system for the functional analyses of human transcription factors and signal transduction pathways such as monitoring e.g. pharmaceutical modifications within the pathway under defined and controlled conditions.In the future, the application of plant cell culture or *Nicotiana benthamiana* as expression systems might further improve the analysis of human proteins in the plant cells.

## Supporting Information

Figure S1 **Subcellular localisation of IκB.** The localization has been analysed in plant protoplasts (A–C) and HEK293 cells (D). (A) shows the subcellular localisation of IκB-ECFP that has been compared with the localization of the nuclear marker Abi5-YFP (B). (C) represents an overlay image of (A) and (B) with additional chlorophyll autofluorescence given in red. In (D) IκB-EYFP can be observed homogenously in cytosol and nucleus of HEK293 cells as both compartments cannot be separated.
(TIF)
