# Supplementary figures and images for: Elements of Transcriptional Machinery Are Compatible among Plants and Mammals
Source: PLoS One. 2013 Jan 11;8(1):e53737. doi: 10.1371/journal.pone.0053737 (PMC3543382; doi:10.1371/journal.pone.0053737)

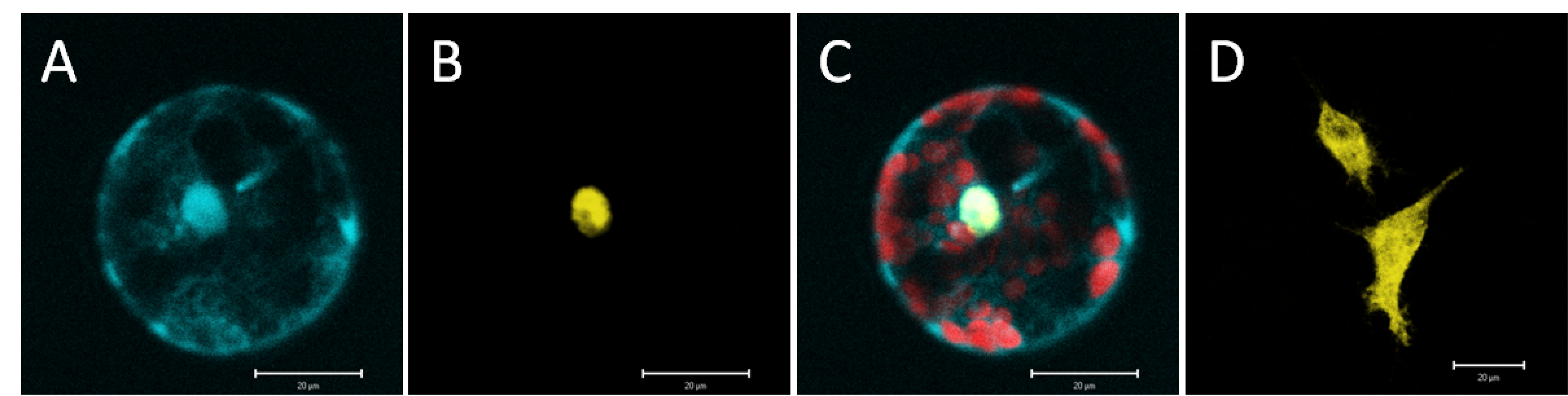

Supplement: Figure S1 — Subcellular localisation of IκB. The localization has been analysed in plant protoplasts (A–C) and HEK293 cells (D). (A) shows the subcellular localisation of IκB-ECFP that has been compared with the localization of the nuclear marker Abi5-YFP (B). (C) represents an overlay image of (A) and (B) with additional chlorophyll autofluorescence given in red. In (D) IκB-EYFP can be observed homogenously in cytosol and nucleus of HEK293 cells as both compartments cannot be separated. (TIF) [file pone.0053737.s001.tif]
